# Obese individuals do not underreport dietary intake to a greater extent than nonobese individuals when data are allometrically‐scaled

**DOI:** 10.1002/ajhb.23743

**Published:** 2022-03-08

**Authors:** Sally P. Waterworth, Catherine J. Kerr, Christopher J. McManus, Rianne Costello, Gavin R. H. Sandercock

**Affiliations:** ^1^ School of Sport, Rehabilitation, and Exercise Sciences University of Essex Essex UK; ^2^ Oxford Brookes Centre for Nutrition and Health Oxford Brookes University Oxford UK

## Abstract

**Objective:**

The aim of this study was to assess the extent of misreporting in obese and nonobese adults on an absolute, ratio‐scaled, and allometrically‐scaled basis.

**Method:**

Self‐reported daily energy intake (EI) was compared with total energy expenditure (TEE) in 221 adults (106 male, 115 female; age 53 ± 17 years, stature 1.68 ± 0.09 m, mass 79.8 ± 17.2 kg) who participated in a doubly‐labeled water (DLW) subsection of 2013–2015 National Diet and Nutrition Survey. Data were log transformed and expressed as absolute values, according to simple ratio‐standards (per kg body mass) and adjusted for body mass allometrically. Absolute and ratio‐scaled misreporting were examined using full‐factorial General Linear Models with repeated measures of the natural logarithms of TEE or EI as the within‐subjects factor. The natural logarithm of body mass was included as a covariate in the allometric method. The categorical variables of gender, age, obesity, and physical activity level (PAL) were the between‐factor variables.

**Results:**

On an absolute‐basis, self‐reported EI (2759 ± 590 kcal·d^−1^) was significantly lower than TEE measured by DLW (2759 ± 590 kcal·d^−1^: F_1,205_ = 598.81, *p* < .001, *η*_*p*_^2^ =0.75). We identified significantly greater underreporting in individuals with an obese BMI (F_1,205_ = 29.01, *p* <.001, *η*_*p*_^2^ =0.12), in more active individuals (PAL > 1.75; F_1,205_ = 34.15, *p* <.001, *η*_*p*_^2^ =0.14) and in younger individuals (≤55 years; F_1,205_ = 14.82, *p* < .001, *η*_*p*_^2^ =0.07), which are all categories with higher energy needs. Ratio‐scaling data reduced the effect sizes. Allometric‐scaling removed the effect of body mass (F_1,205_ =0.02, *p* = 0.887, *η*_*p*_^2^ =0.00).

**Conclusion:**

In weight‐stable adults, obese individuals do not underreport dietary intake to a greater extent than nonobese individuals. These results contradict previous research demonstrating that obesity is associated with a greater degree of underreporting.

## 1 INTRODUCTION

Obesity has become a major challenge to society and places an ever increasing burden on health systems (McLaughlin, Hamilton and Kipping, 2017). The Health Survey for England estimated that the prevalence of obesity (Body Mass Index [BMI] ≥ 30 kg·m^−2^) among UK adults was 28% in 2019, with an additional 36.2% of adults classified as overweight (BMI 25.0–29.9 kg·m^−2^), making a total of 64.2% who are either overweight or obese (HSE, 2019). Obesity prevalence increased steeply between 1993 and 2000, and although the rate of increase has slowed, it has been predicted that by 2030, there could be as many as 11 million more obese adults in the UK (Wang et al., 2011). Obesity results from long‐term energy imbalance; an excess of energy intake (EI) over energy expenditure (Millward, 2013). Accurate assessment of EI is an important aspect of understanding nutrition‐related chronic diseases and plays a significant role in shaping national health guidelines (Boeing, 2013). The National Diet and Nutrition Survey (NDNS) includes self‐reported EI for adults and shows a steady decline in EI from 8.37 MJ·d^−1^ (2000 kcal·d^−1^) in 1987–88 to 7.86 MJ.d^−1^ (1878 kcal·d^−1^) in 2008–11 (Whitton et al., 2011). These figures suggest that the UK population are eating less than previously, which is implausible considering increasing obesity levels. There has been some suggestion that obesity is the result of declining activity levels, but taking into account that heavier individuals require greater amounts of energy to sustain and move their bodyweight, the reduction in activity required to explain the rise in obesity is also too large to be plausible (Millward, 2013). This supports previous authors who question the validity of self‐reported EI (Dhurandhar et al., 2015; Archer, Lavie and Hill, 2018; Archer, Marlow and Lavie, 2018).

A variety of established self‐reported dietary assessment methods exist, including food diaries, 24 h dietary recall and food frequency questionnaires (FFQs). The reliability and validity of these varies between methods and between studies using the same methods in different populations (Rollo et al., 2016). Dietary assessment methods frequently report a range of EIs that are not representative of habitual intakes and are incompatible with survival (Archer, Hand and Blair, 2013). Inaccuracies are further magnified when dietary assessment is conducted in community‐dwelling compared to laboratory environments (Stubbs et al., 2014). Doubly labeled water (DLW) is considered the reference standard method of measuring total energy expenditure (TEE) in community‐dwelling adults (Schoeller & van Santen, 1982). When in energy‐balance, EI is equivalent to TEE (Livingstone & Black, 2003) and DLW can be used as a reference‐standard to validate dietary assessments of EI. Comparing DLW TEE with self‐reported EI has highlighted that inaccurate reporting is prevalent in assessment of EI. A recent systematic review reported that EI is usually underestimated, and inaccuracies range from 11% to 41% for food records, 1%–47% for diet histories and 5%–42% for FFQs (Burrows et al., 2019). Underreporting can be intentional (food being eaten but deliberately not reported and/or food consumption being reduced/changed during the study) or unintentional (food being eaten but genuinely forgotten and/or quantities misjudged) (Garden et al., 2018; Wehling & Lusher, 2019). Magnitude of underreporting is influenced by BMI, sex, age, various social factors, and food types/combinations (Macdiarmid & Blundell, 1998; Rennie, Coward and Jebb, 2007; Stubbs et al., 2014; Gemming & Ni Mhurchu, 2016). Body mass index is a significant predictor of dietary underreporting, with obese individuals underreporting to a greater extent than nonobese individuals (Wehling & Lusher, 2019). It has been postulated that this could in part be affected by obese individuals being more likely to have a negative perception of body image; a greater desire for thinness and being more likely to exhibit periodic dietary restraint (Braam et al., 1998; Macdiarmid & Blundell, 1998; Rennie, Coward and Jebb, 2007; Gemming & Ni Mhurchu, 2016). Whilst these factors have all been reported to influence the magnitude of underreporting, an important consideration is that obese individuals' energy requirements are higher than those of nonobese individuals (Prentice et al., 1996).This is a function of the higher energy cost of moving a larger body, even if obese individuals perform less physical activity or have higher sedentariness than normal weight individuals (Millward, 2013). Obese individuals therefore need to consume greater quantities of energy than normal weight individuals to remain weight stable.

It is possible that the increased underreporting associated with obesity is partly associated with heteroscedastic error (an unequal variance across a range of values) (de Castro, Galea and Bolfarine, 2008), meaning that measurement error in self‐reported intake among obese individuals would be greater due to the larger EI values reported. It is common practice to express energy requirements on a ratio‐scale (relative to body mass) to compare individuals of different body size but this is inappropriate because metabolic cost does not scale in direct proportion with body mass (Heymsfield & Pietrobelli, 2010). Several authors have cautioned against the use of simple ratio‐scaling in adjusting for variation in energy demands and physiological function because this implies that values are proportional over the full range down to zero, and that such expression results in a mathematical error when the linear relationship between variables does not regress through a zero intercept (Tanner, 1949; Weinsier, Schutz and Bracco, 1992; Poehlman & Toth, 1995). Even in cases where a zero‐intercept relationship exists, when data are restricted by age and gender, ratio‐scaling remains problematic due to nonlinearity in the association of energy expenditure with body mass and lean body mass (LBM) (Nevill, Bate and Holder, 2005). Skeletal muscle has a metabolic rate that is nearly three times as high as adipose tissue (~13 kcal·kg·d^−1^ vs. 4.5 kcal·kg·d^−1^ for skeletal muscle and adipose tissue respectively) (Wang et al., 2010). At higher values of LBM, the increase in metabolically active muscle tissue per kg increment of total LBM is reduced compared with that observed at lower LBM values. This results in a curvilnear relationship between LBM and basal metabolic rate (BMR). The shift from a linear to a curvilinear association at high values is greater still when body mass and energy expenditure are scaled at a 1:1 ratio (Weinsier, Schutz and Bracco, 1992; Thomas et al., 2019). Early research suggested metabolic rate scales to the 0.75 power of body mass in humans (West, Brown and Enquist, 1997) or to a power of around 0.66 based on scaling for body surface area (Thomas et al., 2019). More recent data show that BMR scales to weight with a power of 0.64–0.73 (White, Cassey and Blackburn, 2007; Müller et al., 2011). This implies that any measurements of EI or TEE should be scaled allometrically to remove the effect of body mass (Nevill & Holder, 1995). Allometry, in its broadest sense, describes how characteristics of living creatures change with size. Evaluating TEE and self‐reported EI on an allometrically‐scaled basis would control for heteroscedasticity and allow predictors of underreporting to be explored without the confounding effects of body mass.

The aims of this study were (1) to assess the extent of misreporting among obese and nonobese adults participating in the NDNS on an absolute, ratio‐scaled, and allometrically‐scaled basis and (2) to examine factors that may predict misreporting.

## 2 METHODS

The NDNS is a continuous, cross‐sectional survey designed to collect detailed, quantitative information on the food consumption, nutrient intake and nutritional status of the general population aged 1.5 years and over living in private households in the UK (NDNS, 2020). A sub‐section of participants from 2013 to 15 (Study Waves 6–7) were recruited to take part in a DLW study.

## 3 PARTICIPANTS

Data from 221 adult participants (106 male, 115 female) with valid measurements recorded for body mass, BMI, all DLW variables and self‐reported EI were retrieved from the NDNS data repository. All participants provided written informed consent. The study was approved by the Oxfordshire A Research Ethics Committee (#07/H0604/113) and Cambridge South NRES Committee (#13/EE/0016).

## 4 MEASUREMENTS

Measurements have been described in detail elsewhere (Bates et al., 2019) and are summarized below.

### 4.1 Anthropometric measurements

Height and body mass were measured in participants' homes. Briefly, height was recorded to the nearest mm using a portable stadiometer and according to the Frankfort plane method. Body mass was recorded to the nearest 0.1 kg using calibrated scales while participants were wearing light clothing only.

### 4.2 Dietary intake

Self‐reported dietary intake was recorded using a four‐day written food diary. Participants were familiarized with an instruction booklet containing information (including photos of portion sizes, glasses and spoons) and prompts, before being given the opportunity to practice recording. Average nutrient intake and EI were calculated by allocating a food and portion code from the NDNS nutrient databank.

### 4.3 Doubly labeled water

Prior to data collection, a baseline urine sample was collected to establish the abundance of heavy hydrogen (^2^H) and heavy oxygen (^18^O) isotopes in participants' body fluid. Participants were then asked to drink a measured amount of doubly labeled water (^2^H_2_^18^O), proportional to their body mass (80 mg/kg body mass of ^2^H_2_O and 150 mg/kg body mass of H_2_^18^O). Participants collected daily urine samples for the next 10 days, representing about 2.5 half‐lives of peak enrichment. The urine samples were subsequently analyzed for isotopic enrichment by mass spectrometry (^18^O enrichment: AP2003, Analytical Precision Ltd, Northwich, Cheshire, UK; ^2^H enrichment: Isoprime, GV Instruments, Wythenshaw, Manchester, UK or Sercon ABCA‐Hydra 20–22, Sercon Ltd, Crewe, UK). The rate of CO_2_ production was measured, and TEE calculated using Schoeller's equation, with the respiratory quotient taken as 0.85 (Schoeller & van Santen, 1982). BMR was calculated according to Schofield's equations for different age ranges (Henry, 2005). Physical activity level was calculated as the ratio of TEE and BMR.

## 5 DATA ANALYSIS

Total daily EE and self‐reported EI were expressed as absolute values (kcal·d^−1^) and relative to body mass (kcal·kg^−1^·d^−1^). A median split was used to create two age groups (≤55 or ≥ 56 years). Participants were grouped according to obese/nonobese based on BMI > 30 kg·m^2^ and physical activity level based on PAL<1.75 (inactive) or ≥ 1.75 (active), according to WHO guidelines cut‐offs (WHO, 1998).

Descriptive statistics (unadjusted values) are reported as mean ± SD. Underreporting was quantified based on the magnitude of within‐by‐between factor interactions, and on the differences between estimated marignal means (EMMs) and reported by taking anti‐logs of EMMs (EMM ± SE).

Scatterplots of absolute values of TEE against EI showed evidence of greater between‐measure error with increasing values (heteroscedascity). In order to meet the assumption underlying the use of the general linear model (GLM) we took natural logarithms of TEE and EI in our analyses.

To assess whether ratio‐scaling was an appropriate method to remove the effects of body mass, we correlated TEE (Ln[kcal·kg^−1^·day^−1^]) with body mass (Ln[kg]). The association of BM with absolute TEE (r =0.61) was reversed when TEE was ratio‐scaled (r =0.41) confirming ratio‐scaling as an innapropriate method of removing the influence of BMI on TEE.

Misreporting was quantified by calcuating the difference in TEE and EI using full‐factorial GLMs with repeated measures of the natural logarithms of TEE and EI as the within‐subjects factors. Gender, age, obesity, PAL were the between‐factor variables. Method 1 included absolute values as the dependent variables, Method 2 used ratio‐scaled values and Method 3 used allometrically‐scaled values. Values reported are the anti‐logs of estimated marginal means.

The estimated allometric exponent was derived from parameter estimates in the Model 3 GLM (ANCOVA). The allometric Equation Y = a • mass^k1^ • e (where Y is the variable of interest and ‘e’ is the error term) was linearized by taking logs to obtain Ln(Y) = Ln(a) + [k1 • Ln(mass)] + Ln(e). The scaling exponent (k1) was obtained by including Ln(Mass) as a covariate in a general linear model with the difference between LnTEE and LnEI as the dependent variable Ln(Y).

TEE and EI were adjusted for body size allometrically using a multiplicative method with allometric exponents for mass, according to the method of Nevill and Holder (1995). Using GLM with log outcome (TEE or EI) and log scaling factor (mass) as a covariate takes advantage of the fact that the allometric scaling equation can be linearized according to the law of logarithms (Nevill, Bate and Holder, 2005). Nevill et al.'s (2005) method is typically applied using GLM models designed to examine between‐group differences in allometrically scaled outcomes.

Our application of this method to examine differences in within‐group differences (TEE & EI) is appropriate because measures are repeated in the same individual but measures are not simultaneous, so it can be expected that there will be variation in the exact scaling exponent derived from the data. Treating TEE and EI as separate outcome variables by including them as a repeated factor automatically selects the optimal scaling exponent for mass against each of these outcomes, and these are allowed to differ by slope and intercept within the same model. The necessity to speculate the theoretical value of the mass exponent becomes redundant with this approach, since the effect of body mass is effectively eliminated (Albrecht et al., 1993). Nonetheless, to facilitate comparison with data from other studies, we have reported our estimated allometric scaling exponent in the results section (Nevill, Bate and Holder, 2005).

Effect sizes were calculated as Partial Eta Squared (*η*_*p*_^2^). We interpreted values to indicate; small (*η*_*p*_^2^ >0.01), medium (*η*_*p*_^2^ >0.06) or large (*η*_*p*_^2^ >0.14) effect sizes (Cohen, 2013). Statistical significance was set as *p* <.05. All statistical analyses were carried out using Statistics Package for the Social Sciences (SPSS v25, IBM Corp, Armonk, NY).

## 6 RESULTS

The characteristics for the participants are presented in Table 1. Overall, men had a mean age of 54 ± 17 years, mass of 86.0 ± 16.1 kg, height of 1.75 ± 0.06 m and BMI of 28.1 ± 5.0 kg·m^2^ with 34% of men classified as obese according to BMI. Women had a mean age of 53 ± 16 years, mass of 74.2 ± 16.3 kg, height of 1.62 ± 0.06 m. Women's mean BMI was 28.1 ± 5.6 kg·m^2^ with 33% of women classified as obese.

**TABLE 1:** Descriptive statistics (n = 221)

	Male (n = 106)		Female (n = 115)		Obese (n = 73)		Nonobese (n = 178)		Total (n = 221)	
	*Mean*	*SD*	*Mean*	*SD*	*Mean*	*SD*	*Mean*	*SD*	*Mean*	*SD*
Age (years)	54	17	53	16	56	14	52	18	53	17
Stature (m)	1.75	0.06	1.62	0.06	1.68	0.09	1.68	0.09	1.68	0.09
Mass (kg)	86.0	16.1	74.2	16.3	97.5	12.7	71.2	11.5	79.8	17.2
BMI (kg.m^2^)	28.1	5.0	28.2	6.1	34.4	3.9	25.0	3.1	28.1	5.6
Obese (%)	34.0	n = 36	32.2	n = 37					33.0	n = 73
TEE (kcal·day^−1^)	3059	574	2478	454	3000	608	2636	545	2757	591
EI (kcal·day^−1^)	2037	579	1644	474	1801	590	1848	548	1833	561
PAL	1.72	0.24	1.75	0.26	1.70	0.25	1.75	0.25	1.73	0.25

Abbreviations: BMI, body mass index: Obese, BMI > 30 kg·m^2^: TEE, total energy expenditure; EI, energy intake; PAL, physical activity level.

TEE for men (3059 ± 574 kcal·d^−^1) was higher than for women (2478 ± 454 kcal·d^−1^). Self‐reported EI for men (2037 ± 579 kcal·d^−^1) was higher than for women (1833 ± 561 kcal·d^−1^). TEE was higher for younger (≤55 years) adults (2942 ± 595 kcal·d^−^1) than for older (≥56 years) adults (2566 ± 525 kcal·d^−1^) while self‐reported EI was similar (younger 1898 ± 636 kcal·d^−1^, older 1769 ± 467 kcal·d^−1^). TEE for obese individuals (3000 ± 608 kcal·d^−1^) was higher than for nonobese individuals (1636 ± 545 kcal·d^−1^) but self‐reported EI was similar (obese 1801 ± 590 kcal·d^−1^, nonobese 1848 ± 548 kcal·d^−1^). Physical activity levels were similar in obese compared to nonobese individuals (1.70 ± 0.25 vs. 1.75 ± 0.25).

Absolute TEE and EI were positively correlated with body mass (*r* =0.47), as expected. After ratio scaling, energy and body mass were negatively correlated (*r* = −0.55). Body mass explained 30% variance in ratio‐scaled energy and changed the direction of the association, suggesting this is not an appropriate method of scaling for body mass. Our estimated allometric scaling exponent was 0.63 (Equation 1).

(1) $$Y=a•{\mathrm{BM}}^{0.63\;\left(\mathrm{SE}\;0.05\right)}$$

## 7 ENERGY

Absolute EI was significantly lower than TEE (main effects for within‐subjects factors: F_1,205_ = 598.81, *p* <.001, *η*_*p*_^2^ =0.75). Ratio‐scaled EI was significantly lower than TEE (main effect for within‐subjects factors: F_1,205_ = 486.21, *p* <.001, *η*_*p*_^2^ =0.70), suggesting ratio scaling had little influence over the magnitude of the within‐subject difference. When data were allometrically adjusted for body mass, EI was significantly lower than TEE (main effects for within‐subjects factors: F_1,205_ = 11.22, *p* =.001) but the effect size was small (*η*_*p*_^2^ =0.05). Only the allometric‐scaling for body mass reduced the magnitude of within‐group differences.

## 8 SEX

**FIGURE 1:**
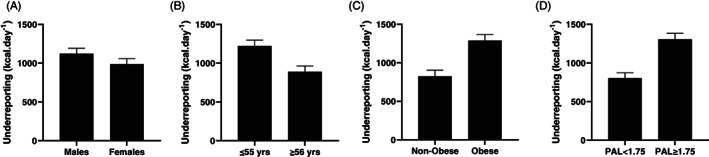
Absolute dietary underreporting (EMM, SE) by (A) sex, (B) age, (C) obese BMI and (D) physical activity level. Values are differences in antilogs of estimated marginal means derived from Repeated‐Measures ANOVA with TEE and EI (kcal·kg^−1^·day) as within‐group effect; Sex, Age, Obesity or PAL included as a between‐group factor

**FIGURE 2:**
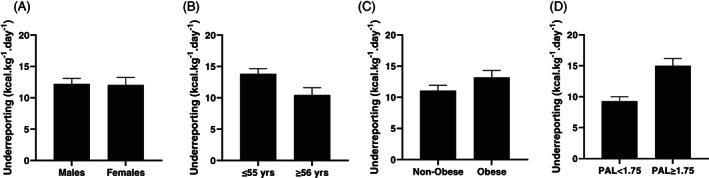
Ratio‐scaled dietary underreporting (EMM, SE) by (A) sex, (B) age, (C) obese BMI and (D) physical activity level. Values are differences in antilogs of estimated marginal means derived from Repeated‐Measures ANOVA with TEE and EI (kcal·kg^−1^·day) as within‐group effect; Sex, Age, Obesity or PAL included as a between‐group factor

**FIGURE 3:**
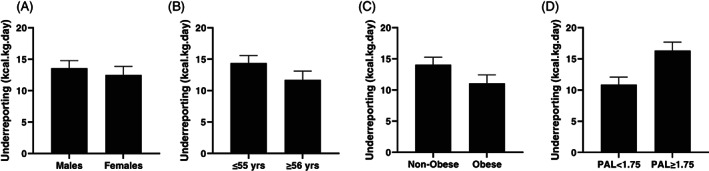
Allometrically‐scaled dietary underreporting (EMM, SE) by (A) sex, (B) age, (C) obese BMI and (D) physical activity level. Values are differences in antilogs of estimated marginal means derived from ANCOVA with TEE and EI Ln(kcal.kg.day) as within‐group effect; Sex, Age, Obesity or PAL included as a between‐group factor and Body Mass Ln(kg) included as a covariate

The absolute within‐by‐between factor interaction for sex was not significant (F_1,205_ =2.51, *p* =0.115 *η*_*p*_^2^ =0.01; Figure 1A). When data were ratio‐scaled, there was no significant within‐by‐between factor effect for sex (F_1,205_ =0.03, *p* =0.865, *η*_*p*_^2^ =0.00; Figure 2A). When data were allometric‐scaling, within‐by‐between factor effect showed females underreported more than males (F_1,205_ =8.71, *p* =.004, *η*_*p*_^2^ =0.04; Figure 3A).

## 9 AGE

There were significant absolutre within‐by‐between factor interactions for age (F_1,205_ = 14.82, *p* <.001, *η*_*p*_^2^ =0.07) with young individuals underreporting more than the older group (Figure 1B). When data were ratio‐scaled, there was a significant, small within‐by‐between factor effect of age (F_1,205_ = 34.15, *p* =.002, *η*_*p*_^2^ =0.05), with younger individuals underreporting more than older individuals (Figure 2B). When data were allometrically‐scaled, there was a significant, small within‐by‐between factor effect of age whereby under 55 s underreported more than older participants (F_1,205_ =7.342, *p* =.007, *η*_*p*_^2^ =0.04; Figure 3B).

## 10 OBESITY

There was a significant, moderate within‐by‐between factor effect of obesity (F_1,205_ = 29.01, *p* < .001, *η*_*p*_^2^ =0.12) with greater overreporting in obese individuals (Figure 1C). When data were ratio‐scaled, there was a nonsignificant small within‐by‐between factor effect of obesity (F_1,205_ =3.69, *p* =0.056, *η*_*p*_^2^ =0.02; Figure 2C). Allometric scaling removed the within‐by‐between factor effect of obesity on underreporting between obese and nonobese participants (F_1,205_ =0.02 *p* =0.887 *η*_*p*_^2^ =0.00; Figure 3C).

## 11 PHYSICAL ACTIVITY LEVEL

There was a large within‐by‐between factor effect for PAL (F_1,205_ = 34.15, *p* < 0.001, *η*_*p*_^2^ =0.14) with active individuals underreporting to a greater extent (Figure 1D). When data were ratio‐scaled, the significant, large within‐by‐between factor effect of PAL remained (F_1,205_ =9.58, *p* < .001, *η*_*p*_^2^ =0.12) with more active individuals underreporting to a greater extent than less active individuals (Figure 2D). When data were allometrically‐scaled, within‐by‐between factor interactions indicated that participants with PAL ≥ 1.75 underreported EI more than participants with PAL<1.75 although the effect size was reduced (F_1,205_ = 18.27, *p* < .001, *η*_*p*_^2^ =0.08; Figure 3D).

## 12 DISCUSSION

This study used a novel approach to assess the extent of misreporting in self‐reported dietary intake in individuals participating in a DLW subset of the NDNS (Years 6 to 7), on an absolute, ratio‐scaled, and allometrically‐scaled basis. We identified significant underreporting of up to 43% in absolute EI. Concurrent with previous literature looking at absolute underreporting (Burrows et al., 2019; Wehling & Lusher, 2019), we identified significantly greater underreporting in individuals with an obese BMI (>30 kg·m^−2^), in more active individuals (PAL ≥ 1.75) and in younger individuals (≤55 years). These were all categories with higher TEE. There were no significant differences in underreporting between males and females (Figure 1). After ratio‐scaling TEE and EI, we identified significantly greater underreporting for more active and younger individuals, with reduced effect sizes. Magnitude of underreporting for obese individuals approached significance (*p* = .056) and had a small effect size (*η*_*p*_^2^ =0.02) (Figure 2). After allometric‐scaling, we identified significant effects of sex, age, and PAL but the effect of obesity on underreporting was removed (Figure 3).

On average, obese individuals underreported EI by 42% of TEE (1287 kcal·d^−1^) compared to underreporting of 31% in nonobese individuals (823 kcal·d^−1^). More active individuals underreported by 41% of TEE (1307 kcal·d^−1^) compared to 31% underreporting in less active individuals (803 kcal·d^−1^). These variables are comparable to previous studies, which have reported that self‐reported EI from food records is 11 to 41% less that TEE measured via DLW in a variety of populations (Burrows et al., 2019). Various explanations for underreporting have been examined, including reduced reporting of ‘socially undesirable’ foods (e.g. highly processed and/or high fat/sugar foods), alteration of self‐reports to project ‘healthier’ behaviors, memory lapses, misrepresentation of portion sizes, high‐eating frequencies and actual changes in feeding behavior when individuals are asked to record their food intake (Braam et al., 1998; Leech et al., 2018; Lichtman et al., 1992; Macdiarmid & Blundell, 1998; Stubbs et al., 2014; Tooze et al., 2004). Authors have not generally considered that reporting errors associated with absolute values might be influenced by the magnitude of the values, as highlighted in this study.

Using DLW is expensive, requires sophisticated laboratory and analytical back‐up and is not readily available, therefore several studies have applied the so‐called ‘Goldberg cut‐off’ to identify potential under reporters (Black, 2000). This cut‐off identifies a minimum plausible energy requirement of 1.55 x BMR, a value corresponding to a normally active (i.e. not sick, disabled or frail elderly) but sedentary population (SACN, 2011). The average PAL in many dietary assessment studies exceeds this, including this study where we identified a mean PAL of 1.73. Using a PAL of 1.55 is conservative, and studies using these (or lower) Goldberg cut‐off values are likely to under‐estimate underreporting, through undetected bias or under‐estimating the degree of bias (Black, 2000). Additionally, many studies use the Schofield equation to estimate BMR. This was developed for a population of normal weight individuals (up to 84 kg) and overestimates BMR in overweight and obese individuals because of their higher proportion of metabolically inert fat mass (Black et al., 1991). Despite the likelihood of under‐estimating the extent of underreporting, studies using Goldberg cut‐offs have identified that at least 12 to 44% of individuals recording food diaries have EIs that are not plausible and are therefore classified as under reporters (Poslusna et al., 2009). Such studies are not able to ascertain the extent of underreporting and might not detect underreporting in individuals with a high PAL. The magnitude of measurement error in self‐reported EI raises doubts about the usefulness of written and memory‐based dietary assessment tools, and adds weight to the suggestion that self‐report measures should not be used to estimate EIs as these appear to have little validity against ‘gold‐standard’ measures (Subar et al., 2015). This has implications for public health recommendations, which have historically relied heavily on self‐reported EI values (Archer, Lavie and Hill, 2018).

Ratio‐scaling is a widely used method of normalizing the results of selected measurements in physiology and clinical medicine. Expressing measurements relative to body mass assumes that the effects of body mass are removed and that values are comparable across different sized individuals (Nevill & Holder, 1995). This practice is less common in dietary intake assessment where absolute values are generally used. After ratio‐scaling TEE and EI, significant main effects remained for age and PAL, with greater underreporting for more active and younger individuals. There was also a nonsignificant trend for obese individuals to underreport to a greater extent, and all effect sizes were reduced. These results were not unexpected because the energy costs associated with a younger age, increased activity and larger BMI are all greater. The larger EIs required to meet larger TEE needs require a higher degree of recording/recall than smaller intakes, therefore using absolute values will increase the magnitude of misreporting purely because larger values have higher associated errors. For example, underreporting by 20% for a person with a TEE of 2000 kcal·d^−1^ would be 400 kcal·d^−1^, whereas for a person with a TEE of 4000 kcal·d^−1^, this would double to 800 kcal·d^−1^. Ratio‐scaling (expressing measurements relative to body mass) aims to control for this but overcompensates for weight as metabolic rate does not increase proportionally with body mass (Heymsfield & Pietrobelli, 2010). Our results support this as we showed that ratio‐scaling TEE and EI did not remove differences due to body mass (Figure 2C). The use of an allometric scaling exponent is therefore warranted to overcome the effect of heteroscedasticity and remove the effect of body mass on measures of TEE and EI.

Allometric‐scaling exponents for metabolic rate have been the subject of many reviews, and proposed scaling exponent values for body mass have ranged from 0.64 to 0.73 (White, Cassey and Blackburn, 2007; Müller et al., 2011). These body mass exponents are associated with several assumptions therefore we calculated the specific body mass scaling exponent for the present dataset as 0.63 with a SE of 0.05 (Equation 1). The 95% CI of this mean estimate of the exponent (0.57–0.77) sit within previously reported values. TEE comprises BMR and energy expenditure related to thermogenesis and exercise, which can be significantly higher than resting energy expenditure. Maximum metabolic rate (V̇O_2max_) also has an exponent that lies somewhere between these values (Lolli et al., 2017). Results showed that allometric‐scaling removed the effect of obesity on underreporting (*p* =0.887), indicating that obese individuals do not underreport to a greater extent than nonobese individuals when the effect of their larger body mass and associated greater energy needs are removed (Figure 3C). Previous research has investigated reasons for underreporting in obese individuals to identify effective techniques to reduce this in clinical practice (for a review see Wehling & Lusher, 2019), but few researchers have considered that obese individuals have larger energy requirements. The more extensive underreporting seen in obese individuals might therefore simply be a function of larger EI values and associated measurement errors. This observation is supported by a significant main effect of PAL, as more active individuals also have greater energy requirements, regardless of body size (Figure 3D). Future research should therefore consider factors that drive a high EI rather than factors that predict underreporting in obese individuals, as absolute values for underreporting have inherent limitations.

Despite obese participants' higher absolute TEE (approx. 400 kcal·d^−1^), both groups reported similar EI (Table 1). This observation is in agreement with previous research which has shown that obese individuals consistently reported consuming the same or less energy than their normal weight counterparts (Lichtman et al., 1992; Myers et al., 1988). Given TEE is equal to EI in weight‐stable individuals, this suggests that regardless of what individuals actually ingest, they report consuming the same. People can have different motives and reasons for underreporting EI, of which some are intentional and others nonintentional (Connor, 2020). A widely‐held assumption is that obese individuals intentionally under‐report by a greater degree than nonobese individuals to improve their self‐esteem as a form of self‐deception or self‐presentation because they want to present themselves in a positive light to others (Wehling & Lusher, 2019). Self‐reported absolute EI was similar for obese and nonobese individuals and was significantly lower than national dietary recommendations. Mean self‐reported EI was 2057 kcal·d^−1^ in males and 1599 kcal·d^−1^ in females, compared to guidelines of 2500 kcal·d^−1^ and 2000 kcal·d^−1^ for males and females respectively (PHE, 2018). Nutrition guidelines provide individuals with an anchor point, and it is known that anchoring is a process than can influence decisions and behaviors (Chapman, Johnson and Chapman, 2002). Accurate estimation of energy content is difficult and compounded by the wide availability of inexpensive, nutrient‐poor and energy‐dense foods in the current food environment which are heavily marketed, not clearly labeled and served in large portions (Roberto & Kawachi, 2014). Research has argued that numeric judgments made under uncertain conditions are easily influenced by readily available anchors (Gigerenzer & Gaissmaier, 2011), such as the national nutrition guidelines. Individuals in this study, however, reported lower EI than guideline targets. Reasons for this warrant further investigation but might be partly due to a desire to be a lower body weight and knowledge of the cost and burden of overweight and obese individuals (Connor, 2020). Given the magnitude of the discrepancies between TEE and EI in this and previous studies, it is questionable whether self‐reported EI should be used to guide dietary prescription and public health guidelines. Future research might be better focusing on dietary risk factors for obesity, such as foods with high‐energy density, foods with poor satiation (e.g., ultra‐processed foods), high‐fat low‐fiber foods and sugary beverages, all of which drive a high EI (Fogelholm et al., 2012). This aligns with the concept of dietary patterns, a move to comprehensively represent the totality of the diet and nutrient profiles by placing emphasis on foods and beverages rather than individual nutrients or single foods (Jacques & Tucker, 2001). Dietary patterns are becoming established as providing more useful insights into associations of diet with health outcomes, including obesity, compared to individual dietary components (Johnson, Toumpakari and Papadaki, 2018).

This study is not without its limitations, which are important for the reader to consider when interpreting the results shown herein. Data are drawn from a sub‐sample of the NDNS and may not be representative of the general or current population. The mean area‐level deprivation score for the sample was low, indicating participants lived in relatively affluent areas. Participants were voluntarily participating in a dietary survey; therefore, the sample may have been biased towards health‐conscious individuals. Self‐reported dietary intake was recorded 2–3 weeks prior to DLW measures. Participants reported that they remained weight‐stable, were not dieting, and had not changed eating behaviors but this was not verified. Food diaries were hand‐written which may have been more challenging for younger adults, given the more widespread use of computers in younger generations. Our scaling exponent was estimated for TEE because this was measured using DLW and mesures were deemed more trustworthy than EI. Despite being the gold‐standard for TEE, DLW relies on a number of assumptions and has its own inherent error which can be up to 8% (Butler et al., 2004; Ravelli & Schoeller, 2021). Finally, participants were aware of the DLW study which might lead to unrepresentative behavior during the study period including changes in eating behaviors to reduce EI and increased activity levels to increase TEE (van Sluijs et al., 2006).

In conclusion, our novel approach of allometrically scaling TEE and EI to remove the effects of body mass showed that in weight‐stable adults, obese individuals do not underreport dietary intake to a greater extent than nonobese individuals. This contradicts previous research that has reported obesity is associated with a greater degree of underreporting. The high‐absolute errors in self‐reported EI and the effect of body mass on TEE and EI raises questions as to the usefulness of written dietary records. Future research should investigate factors that drive a high EI rather than factors that affect underreporting, given the large disparity between actual and self‐reported EI.

## CONFLICT OF INTEREST

The authors declare there are no potential conflicts of interest.

## AUTHORS CONTRIBUTION

All authors formulated the research question and designed the study. Gavin RH Sandercock and Sally P Waterworth analyzed the data and interpreted the findings. Sally P Waterworth drafted the manuscript. All authors made significant contributions to editing and finalizing the manuscript.

## Data Availability

The data that support the findings of this study are openly available in the UK Data Service Repository at http://doi.org/10.5255/UKDA-SN-8405-1, Study Number (SN) 8405 and http://doi.org/10.5255/UKDA-SN-6533-16, SN 6533.
